# Femtosecond multimodal imaging with a laser-driven X-ray source

**DOI:** 10.1038/s42005-023-01412-9

**Published:** 2023-10-11

**Authors:** Adam Doherty, Sylvain Fourmaux, Alberto Astolfo, Ralf Ziesche, Jonathan Wood, Oliver Finlay, Wiebe Stolp, Darren Batey, Ingo Manke, François Légaré, Matthieu Boone, Dan Symes, Zulfikar Najmudin, Marco Endrizzi, Alessandro Olivo, Silvia Cipiccia

**Affiliations:** 1 Department of Medical Physics and Biomedical Engineering, University College London, 2 Malet Pl, London, WC1E 7JE UK; 2 Institut National de la Recherche Scientifique—Énergie, Matériaux et Télécommunications, Université du Québec, 1650 Lionel Boulet, Varennes, J3X 1P7 QC Canada; 3 Helmholtz-Zentrum Berlin für Materialien und Energie Hahn Meitner Platz 1, 14109 Berlin, Germany; 4 The John Adam Institute for Accelerator Science, Imperial College London, Prince Consort Road, South Kensington, London, SW7 2BW UK; 5 Central Laser Facility, Rutherford Appleton Laboratory, Harwell Campus, Didcot, OX11 0QX UK; 6 UGCT-RP, Department of Physics and Astronomy, Ghent University, 9000 Ghent, Belgium; 7 Diamond Light Source, Rutherford Appleton Laboratory, Harwell Campus, Didcot, OX11 0QX UK

**Keywords:** Applied physics, Plasma physics

## Abstract

Laser-plasma accelerators are compact linear accelerators based on the interaction of high-power lasers with plasma to form accelerating structures up to 1000 times smaller than standard radiofrequency cavities, and they come with an embedded X-ray source, namely betatron source, with unique properties: small source size and femtosecond pulse duration. A still unexplored possibility to exploit the betatron source comes from combining it with imaging methods able to encode multiple information like transmission and phase into a single-shot acquisition approach. In this work, we combine edge illumination-beam tracking (EI-BT) with a betatron X-ray source and present the demonstration of multimodal imaging (transmission, refraction, and scattering) with a compact light source down to the femtosecond timescale. The advantage of EI-BT is that it allows multimodal X-ray imaging technique, granting access to transmission, refraction and scattering signals from standard low-coherence laboratory X-ray sources in a single shot.

## Abstract (web summary)

Laser-plasma electron accelerators are ultracompact and come with an embedded betatron X-ray source with small source size and ultrashort pulse length. The authors combine the edge illumination-beam tracking technique with a compact plasma X-ray source and present a demonstration of multimodal imaging down to the femtosecond timescale.

## Introduction

**Fig. 1:**
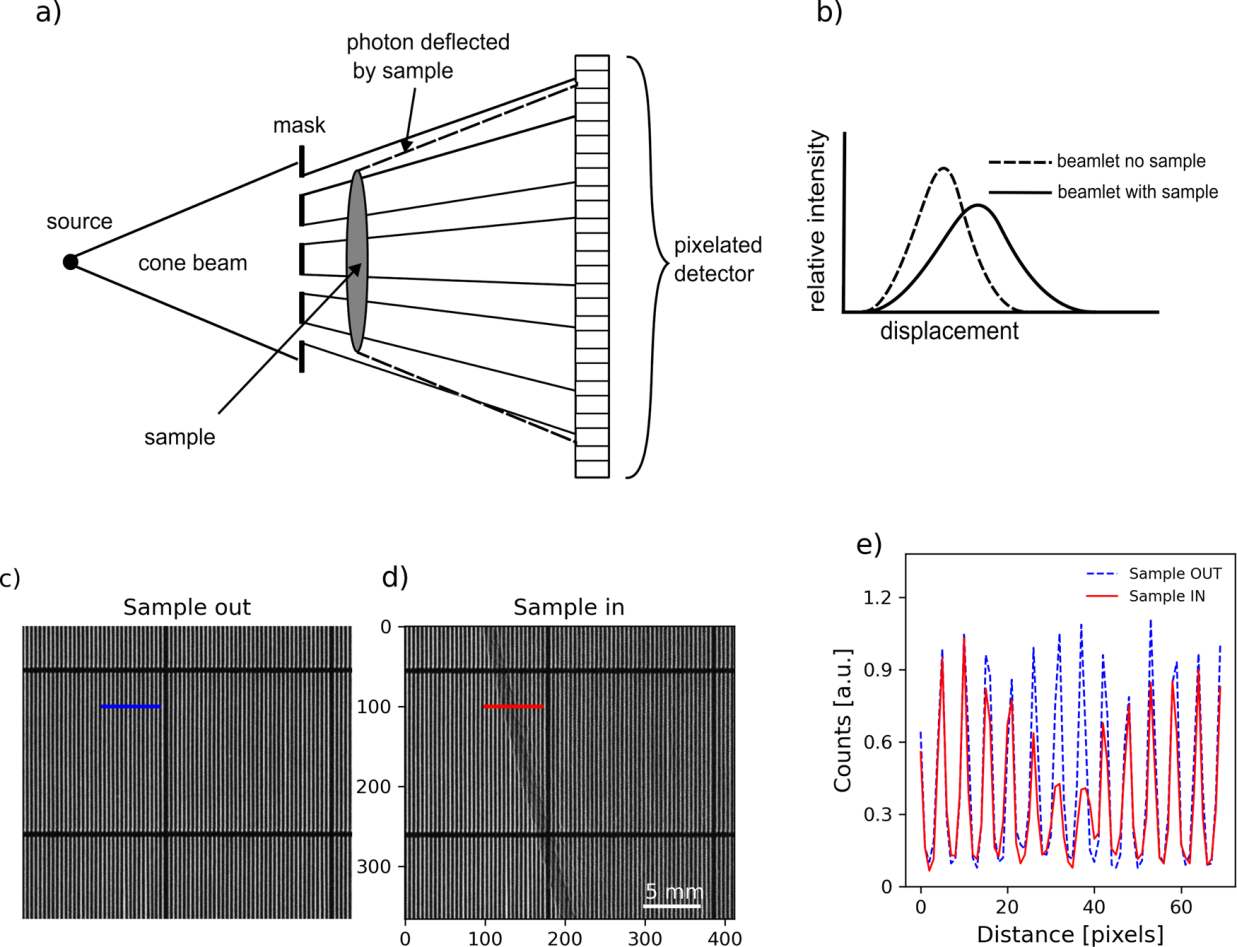
Edge illumination principle. **a** An absorbing mask splits the X-ray beam into beamlets which are imaged with a pixelated detector. **b** the presence of the sample between the mask and the detector changes the beamlets’ shape. **c**, **d** Cropped detail (2.7 × 3.1 mm) of transmission images of the mask used in this experiment without (**c**) and with (**d**) sample (detail of a leaf margin). **e** line profiles from **c** and **d** of the beamlets with (solid red line) and without sample (dashed blue line). See Supplementary Note 1 Fig. S1 for the full-size raw images.

X-ray imaging is one of the most widely used non-destructive inspection techniques with applications in industry, research, security, and healthcare. Conventional X-ray imaging is based on the absorption of X-rays by the inspected object. It consists of looking at the shadow cast by the sample and therefore it is not suitable to image weakly absorbing specimens or to distinguish between different parts of a specimen with similar absorption properties. X-ray phase-contrast imaging (XPCI) is based on X-ray refraction, where the phase changes induced by the sample translate into the deviation of the X-ray paths. The deviation, or refraction angle, is proportional to the first derivative of the phase change. X-ray refraction is the dominant mechanism of interaction for weakly absorbing specimens. Samples or sample details invisible to X-ray absorption imaging become visible using XPCI. Various XPCI methods have been implemented in recent years, from free-space propagation^1^, to grating interferometry^2^ and speckle imaging^3,4^. Edge illumination^5^ (EI), based on the inspection of a sample through multiple beamlets created by a pre-sample mask, has lower X-ray beam quality requirements (in terms of coherence) than other methods, which has made its translation to standard laboratory X-ray sources easier. A comprehensive review of the EI method can be found in ref. ^6^. A simplified implementation of EI, called beam tracking method^7^ (EI-BT), is shown in Fig. 1. The EI-BT method has two main advantages with respect to “classic” EI. The first is a simpler setup: EI requires two masks, one before the sample and one in front of the detector, which need to be aligned with respect to each other, while EI-BT requires only one mask, with no particular alignment requirements. The second advantage is that EI-BT allows to access the transmission, refraction, and scattering channels in a single shot, while two-mask EI requires scanning one mask with respect to the other. The downside is the more stringent detector requirement in EI-BT with respect to two-mask EI. In EI the aperture of the detector mask effectively determines the pixelation, and as a result, it allows the use of much larger detector pixels, regardless of whether direct or indirect detection methods are used. In EI-BT, following beam shaping by a pre-sample mask, the detector must have a pixel size small enough to resolve the individual beamlets directly, usually a factor of 1:5 with respect to the magnified mask period. Transmission, refraction, and scattering properties of the sample can be retrieved simultaneously from the changes in the beamlets: a variation in intensity corresponds to sample transmission, a shift of the beamlet centre corresponds to refraction and a broadening of the beamlets corresponds to scattering. The latter, also known as dark-field imaging, is caused by features smaller than the system’s spatial resolution. This channel has been proven extremely effective for detecting the early formation of cracks in various materials or nucleation sites in additive manufacturing processes^8^. The resolution of the technique is determined by the period of the mask. However, by scanning (dithering) the sample (or the mask) across a period, the resolution increases and becomes equal to the largest between the mask aperture and the dithering step^9,10^. Therefore, the single mask position approach has the advantage of being compatible with single-shot applications, while the scanning (dithering) version enables higher resolutions. EI and EI-BT have proven their robustness in a wide range of applications from intra-operative specimen imaging^11^ to security inspections^12^ using laboratory sources.

Laser-wakefield accelerators^13^ (LWFA) are electron accelerators based on the nonlinear interaction of a high-power laser and a plasma. The working principle is simple: a high-intensity laser pulse travelling through plasma forms a plasma wave. The plasma electrons surfing the wave can be accelerated to relativistic energies (GeV) in a few millimetres. The compactness of this accelerating structure is formidable, up to 1000 times smaller than standard radiofrequency cavities. LWFAs^14–16^ have triggered a revolution in the field of particle accelerators and inspired the design of novel acceleration schemes^17^. On top of their compactness, LWFAs have another remarkable feature: they incorporate a bright X-ray source, with femtosecond pulse duration, also known as betatron radiation^18^. The emission process is due to the plasma acting like a wiggler and driving transverse oscillations of the electrons during the acceleration. The betatron source is a few microns in size, the emission has a synchrotron-like energy spectrum. The pulse duration of the betatron source is on the order of the laser pulse duration thus providing femtosecond resolution. This is a very competitive approach compared to large-scale infrastructures including X-ray free electron lasers (XFELs)^19^ or slicing at synchrotron facilities^20–22^. The source size and ultra-short pulse duration make the betatron radiation one of the brightest compact light sources^23,24^, which has enabled several successful X-ray imaging applications^25–30^, including radiography of batteries^31^, high-resolution phase-contrast imaging^32^ and single-shot femtosecond phase-contrast imaging^33,34^.

To best harness the distinctive properties of the betatron radiation, it is paramount to focus on its time resolution. There is the need to combine this source with imaging methods which encode multiple information, including and beyond transmission and phase, into a single-shot acquisition approach.

In this work, we present a first step to meet this need. Here, we bring together the single-shot multimodal capability power of the EI-BT technique and the unique features of the betatron X-ray source. We simultaneously retrieve absorption, phase and dark field images of test samples and battery materials. We then prove that three-channel imaging in single-shot femtosecond mode is possible. We discuss how the results open a new range of imaging applications for betatron sources, for example following ultrafast dynamic processes and enabling pump-probe experiments.

## Results and discussion

**Fig. 2:**
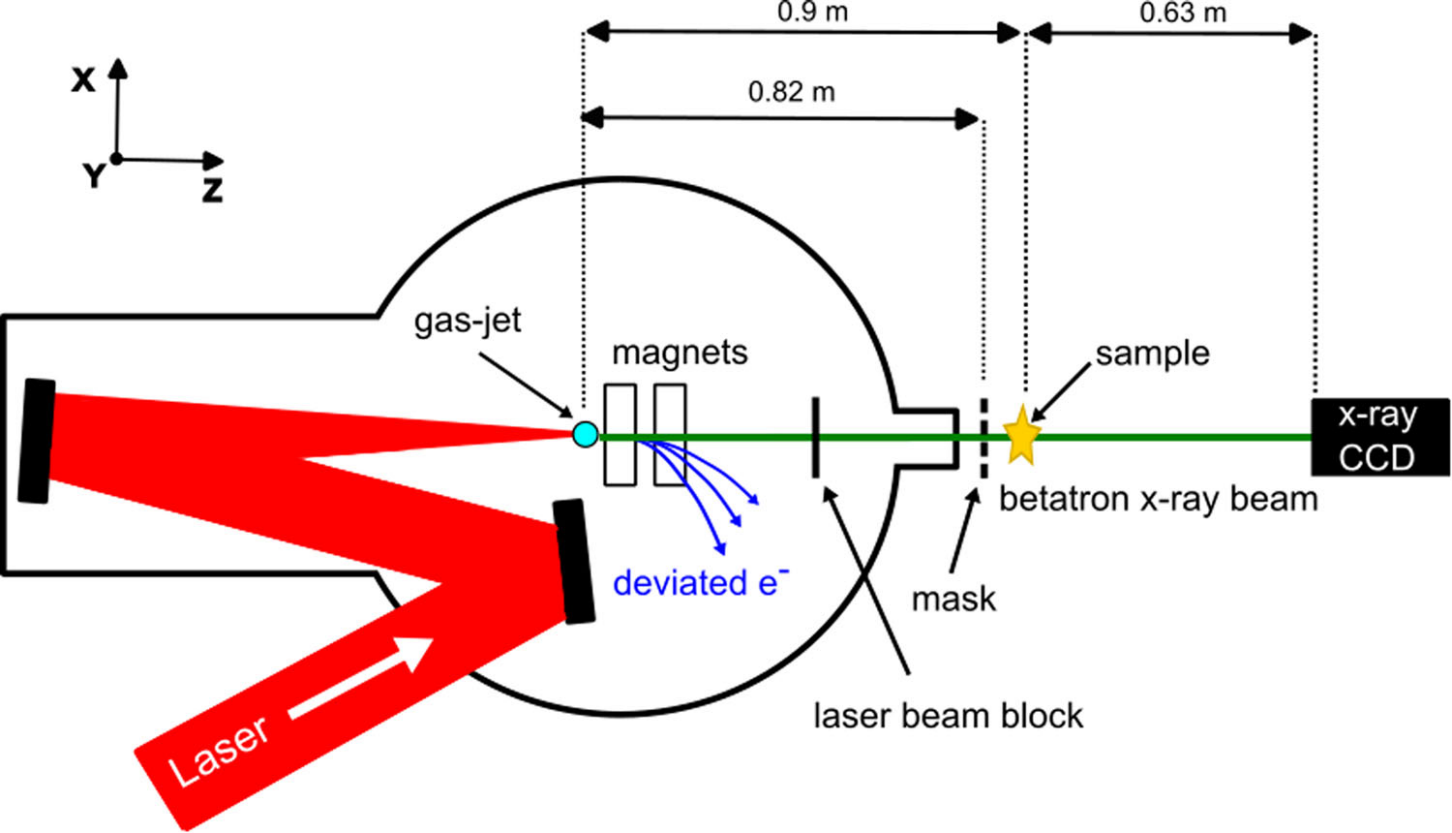
Schematic of the experiment at ALLS. The laser is focused onto a gas jet inside the interaction chamber. The accelerated electrons are swept away by magnets after the gas jet. The laser is stopped by an appropriate beam-block, while the X-ray propagates outside the chamber. The mask is placed after the chamber exit window, the pixelated X-ray detector just downstream. The sample is mounted between the mask and the detector.

The experiment was performed at the advanced laser light source (ALLS) facility at INRS-EMT in Varennes (Qc, Canada). The schematic of the experiment is shown in Fig. 2.

A Ti:sapphire laser produces 3.2 J onto a target with a pulse duration of 22 fs FWHM corresponding to 145 TW and a linear polarisation. The laser pulse was operated at 0.5 Hz and focused onto a supersonic He (99%)/N (1%) gas jet producing an electron density of 3.5 × 10^18^ cm^−3^. The resulting electron beam of ~ 0.7 nC and average energy ~300 MeV, is diverted from the laser axis using a set of permanent magnets^35^ (see Supplementary Note 2 for further details). The laser-beam is blocked using a 100 μm glass plate and a 20 μm Al foil placed after the magnets. The betatron radiation exits the vacuum of the interaction chamber passing through a 250 μm beryllium window. An average of 5 × 10^9^ photons sr^−1^ per 0.1%BW per shot with a 26 mrad divergence at FWHM is produced. This corresponds to a total of 2 × 10^9^ photons per shot on average.

The EI-BT setup was assembled just outside the exit window of the interaction chamber. The mask consisted of long parallel slits (forming a 1D vertical grating) with 39 μm period and 12 μm apertures in a 50 μm thick tantalum foil. Horizontal bridges across the grating, vertically spaced 1.5 mm are added to provide mechanical strength to the free-standing structure. The mask was installed on a three-axes Smaract motor stack (x, y and roll) at 0.82 m from the source. The sample was mounted on a 2-axes (x, y) Smaract motor stack at 0.9 m from the source. The detector, an Andor IKON-M BR-DD with 1024 × 1024 13 μm pixels was placed 1.53 m from the source, which corresponds to a mask magnification of 1.8 and a sample magnification of 1.7.

**Fig. 3:**
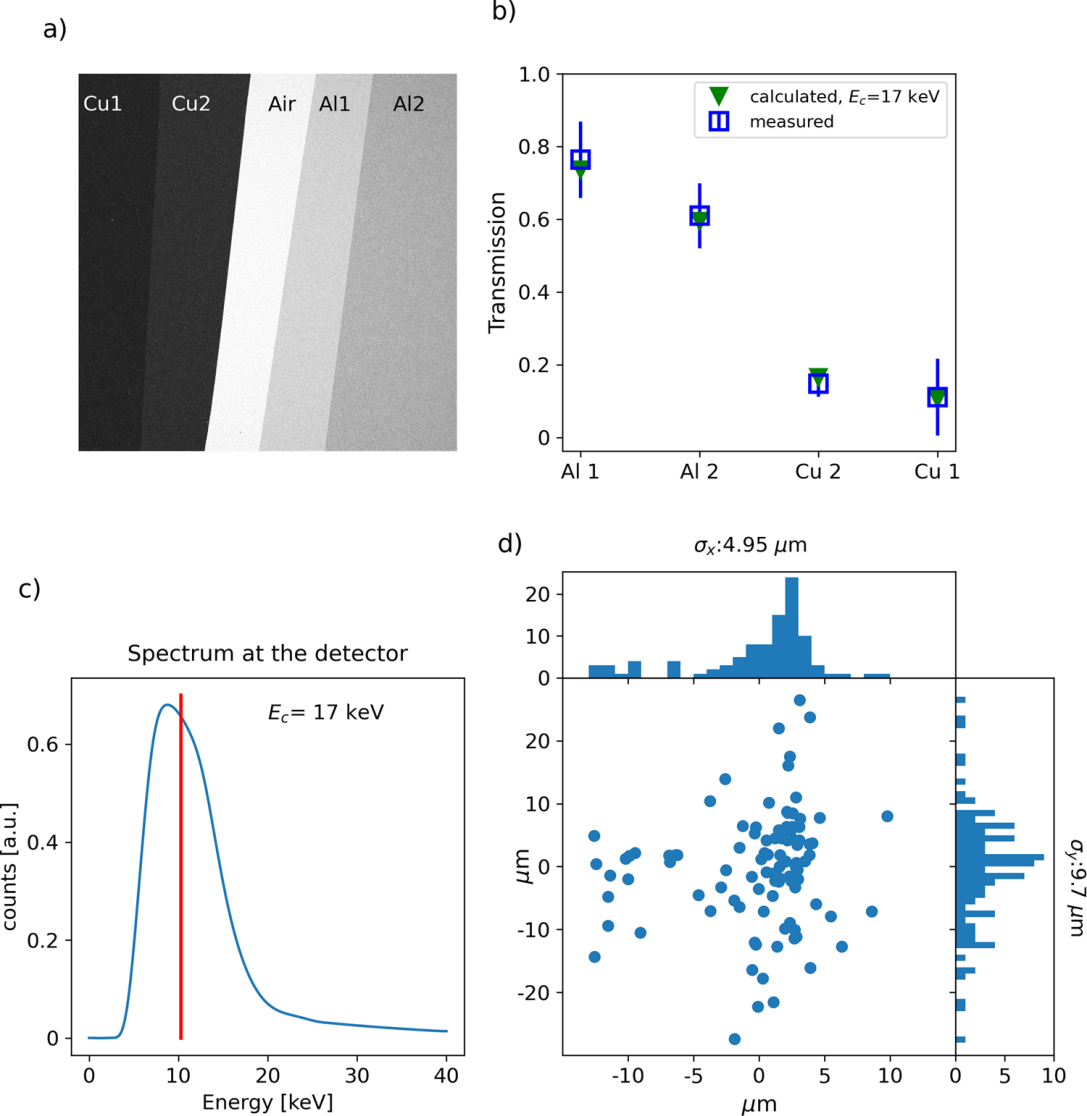
Source characterisation. **a** Transmission image of the X-ray beam through a set of metal filters as recorded by the detector: transmission through 136 and 68 μm of copper (Cu1 and Cu2) and through 50 and 100 μm of aluminium (Al1 and Al2) are shown. **b** The transmission is compared to that of a synchrotron-like spectrum with 17 keV critical energy. The error bars indicate the standard deviation. **c** Calculated X-ray spectrum for a critical energy of 17 keV as seen at the detector: the laser beam-stop, exit window and air before the detector absorbs the low energy part of the spectrum. The average energy of 10.3 keV is marked with the vertical red line. **d** Source position fluctuation obtained from 100 consecutive shots. See Supplementary Note 3 for further information.

We characterised the X-ray energy by looking at the transmission through a set of metal filters placed in front of the Andor camera. The average over 100 shots of the transmission through the filters (placed side by side to speed up the procedure) recorded by the detector is shown in Fig. 3a. The average transmission values calculated by considering the camera efficiency and the effect of the laser beam-block and the Be window correspond to a critical energy of 17 keV (Fig. 3b), where the critical energy is defined as the energy that split the power spectrum in half. The fluctuation of the critical energy over 100 shots was measured to have a standard deviation of 2.3 keV. The calculated spectrum as seen by the detector, which is corrected for the absorption of the laser beam block, exit window and air, has an average energy of 10.3 keV (see Fig. 3c).

We acquired the reference image for the beamlets (mask in, sample out) by averaging 100 individual shots. By registering the mask movement over 100 shots, we measured the X-ray source position fluctuation^36^ (Fig. 3d) in the vertical and horizontal direction to obtain a standard deviation of 9.7 and 4.95 μm, respectively.

**Fig. 4:**
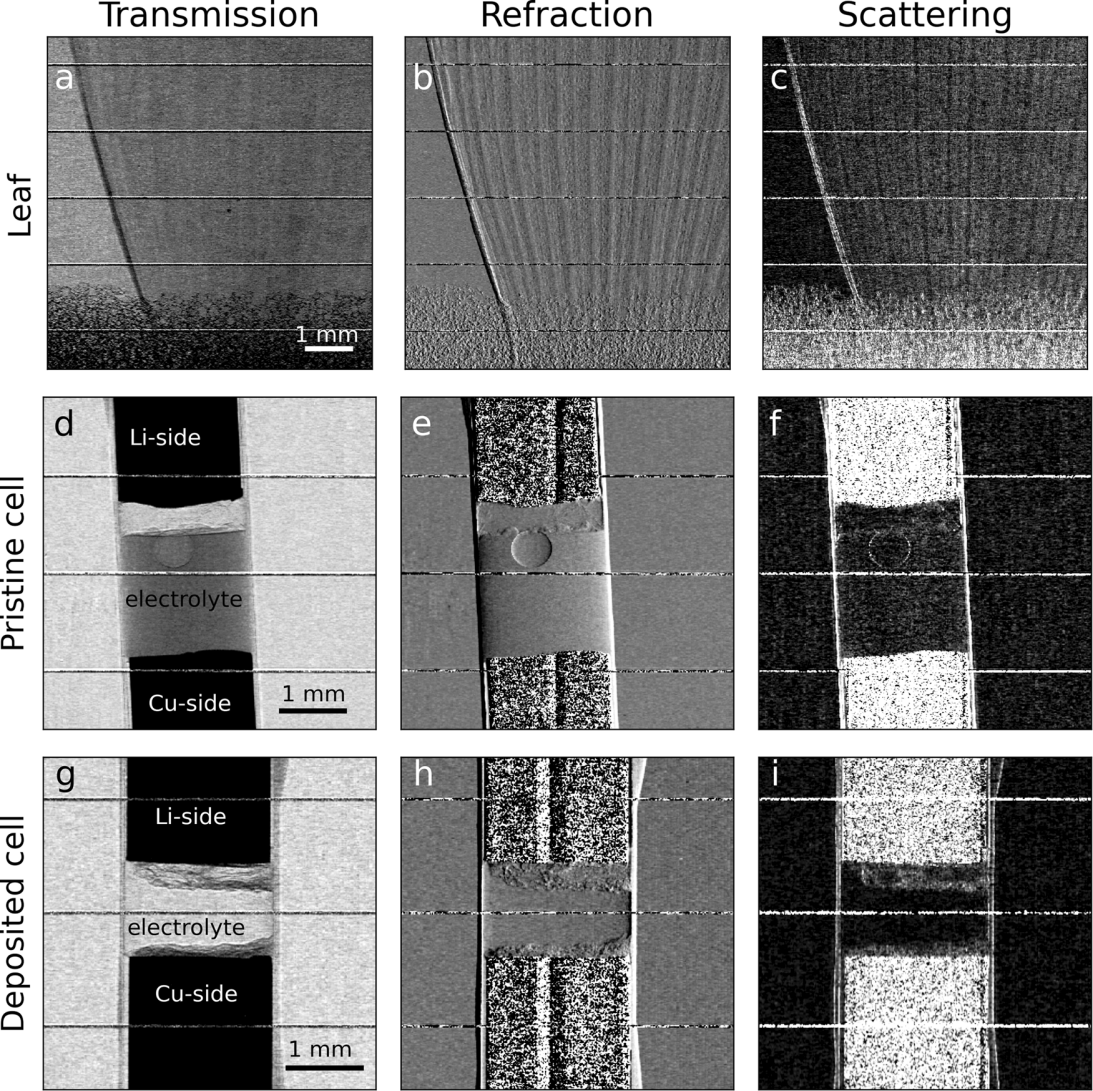
Retrieved multimodal images. Transmission, refraction, and scattering were retrieved for a leaf (**a**–**c**) and two electrochemical samples (pristine, **d**–**f**, and after electrochemical deposition, **g**–**i**). The horizontal lines present in all images are artefacts due to horizontal bridges that interrupt the vertical grid every 1.5 mm to reinforce the mechanical strength of the free-standing mask. Avoiding a substrate minimises photon loss by absorption.

The EI-BT acquisition was performed by dithering (stepping) the sample across the structured illumination in four different positions at steps of 10.7 μm, which corresponds to the mask pitch (39 μm) divided by four (the number of dithering steps) times the sample-mask magnification (1.1). For all results presented below, 100 images were acquired and averaged at each of the four dithering positions, corresponding to a total of 400 shots per sample (for the raw images of the mask with and without sample see Fig. 1c, d). We imaged a leaf on a foam base, and two electrochemical cells (for the cells preparation procedure see methods). The first cell is imaged pristine (before lithium electrodeposition on the copper-metal side), and the second after electrochemical deposition of lithium on the opposite copper-metal electrode, in the form of dendritic or platted lithium^37^. This deposition is one of the causes of battery failure and is therefore an important mechanism to be understood and prevented. The three channels, transmission, refraction, and scattering were retrieved, with the results shown in Fig. 4. For all images, the horizontal pixel size is determined by the dithering step and corresponds to 10.71 μm, the vertical pixel size is given by the pixel size of the camera,13 μm, multiplied by the binning factor, x2, and divided by the magnification, x1.7, which corresponds to 15.3 μm. In Nyquist terms, this means that for all the dithered acquisitions the spatial resolution is limited to 21.4 μm horizontally and 30.6 μm vertically.

The complementarity of the three retrieved channels is apparent in all imaged samples. The leaf veins, barely visible in the transmission image, are evident in the refraction image, while the leaf margin and the smaller (sub-resolution) veins within the leaf lamina generate a detectable scattering signal, as does the foam at the bottom of the image. The maximum recorded scattering angle was measured to be of just above 12 μrad. Assuming that multiple scattering is negligible, and an average X-ray energy of 10.3 keV, we estimate the smallest features contributing to the scattering signal to be below 5 μm.

Likewise, in the pristine electrochemical cell, transmission shows the lithium layer on the Li-side (the top part of the cell in Fig. 4) and the electrolyte between the Li- and the Cu-side (the bottom part of the cell in Fig. 4), with a trapped argon bubble.

Details of the bubble and the lithium-electrolyte boundary are evident in the refraction image, while the scattering image shows only a weak signal at the lithium-electrolyte boundary and at the bubble edge. However, these signals are very different for the lithium-deposited cell. In all the retrieved images, we can notice a lithium stratification on the Cu-side and changes on the Li-side. Contrary to the pristine cell, the structural changes are most visible in the scattering channel.

**Fig. 5:**
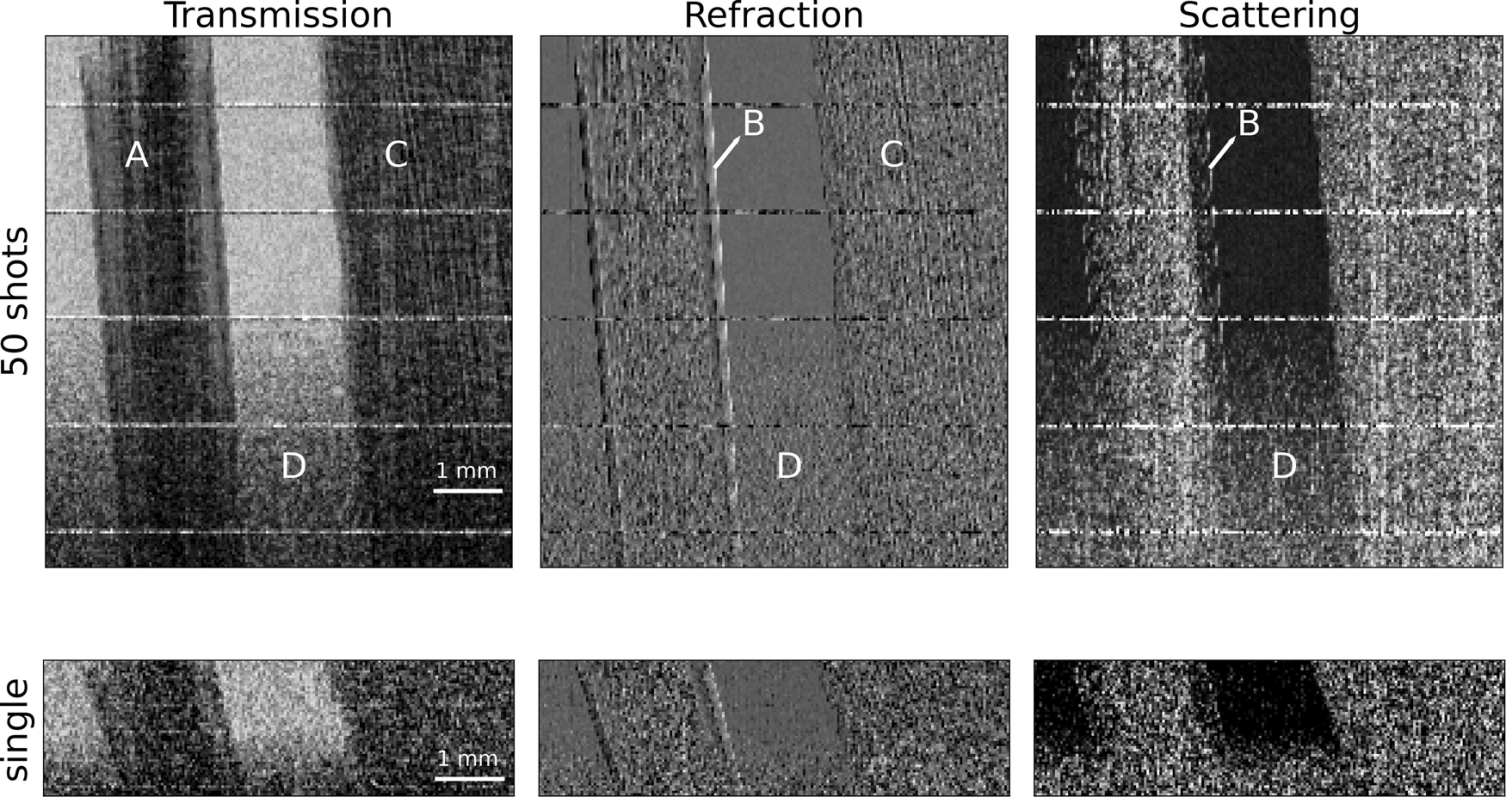
From multi to single-shot imaging. A sample made of carbon fibres (**A**) wrapped in Kapton tape (**B**), bamboo wood (**C**) on the top of a foam layer (**D**) was imaged, acquired without dithering, and retrieved by decreasing the number of cumulated images from 50 to single shot. The field of view is 7.3 (H) x7.8 (V) mm. The vertical binning is 2 and 8, respectively, for the 50 and single-shot images.

In addition to the multi-shot dithered acquisitions presented above, we imaged a bamboo wood and a carbon fibres sample on a foam base without using dithering. Figure 5 shows the three retrieved channels for 50 shots for the single-shot mode. The 50-shot image corresponds to a photon flux of the order of 2.5 × 10^6^ photons/mm^2^ recorded by the detector. The horizontal pixel size is defined by the mask pitch, which is 39 μm. To partially compensate for the lower statistics, the raw images acquired in single-shot mode were binned x4 with respect to the 50-shot ones, which causes the single-shot images to be vertically compressed. Again by taking the Nyquist-Shannon sampling theorem into account, the spatial resolution for the single-shot images is 78 μm horizontally and 122.4 μm vertically. Despite some loss of details due to the vertical binning and the lower statistics, the complimentary information carried by the three channels, is noticeable, e.g., in the distinctive features of the Kapton wrapped around the carbon fibres (in the refraction and scattering channels) and the bamboo wood stripy structure (in transmission and refraction), are still visible in the femtosecond single-shot image.

In this first proof-of-concept experiment, we have demonstrated that three-channel, multimodal imaging is possible both in multi-shot mode and, at lower resolution, in single-shot at the femtosecond: the first proves the stability of the source, the second allows the harnessing of one of its most distinctive features, namely the femtosecond pulse duration. We opted for a mask with a relatively large aperture size and pitch (12 and 39 μm, respectively), to allow for sufficient photon statistics to be collected. This choice allowed us to obtain the first demonstration of single-shot femtosecond multimodal imaging. However, to achieve higher-quality single-shot imaging, more flux is required. This will allow for an increase in resolution, as the reduced statistical noise would eliminate the need for vertical binning. It will also enable the use of smaller period masks: high aspect ratio, shorter pitch and smaller aperture masks, e.g., 7 μm period and 1.5 µm aperture, have been successfully manufactured and used in EI-BT imaging before^38^.

There are several areas where improvements are possible toward higher flux, specifically laser parameters, plasma profile and detector. More specifically, the ALLS laser, currently operating at 145 TW, is expected to be operated at its full 750 TW power in the near future. This will correspond to an increase in energy and charge of the electron beam and consequently higher X-ray photon flux and energy. Following the scaling from Kneip^39^, the photon flux is proportional to *P*^*2/3*^ where *P* is the laser power; therefore, a factor of 3 increase in flux is expected when running ALLS laser at full power. Concerning the plasma profile, in the proposed experiment we used a gas jet with a flat-top density profile. It has been recently proven that the use of a tailored plasma density can substantially enhance the betatron emission by upshifting the critical energy by one order of magnitude and increasing the photon flux in the hard X-ray region (>10 keV) by up to two orders of magnitude^40^. Finally, lower noise integrating detectors such as hybrid-pixel-detectors^41^ could provide a higher detection efficiency, although their typically larger pixel may impose a higher magnification setup (>x10). The combined effect of all these elements would provide a flux increase of a factor of 100, which would make single-shot multimodal imaging achievable at much higher resolutions.

Regarding future development, of particular interest is the ability to perform EI-BT at higher X-ray energies. EI has been successfully implemented 120 kVp^12^, with standard laboratory sources and at 85 keV^42^ and more recently at 120 keV^43^ at synchrotrons. While limits will still exist related to penetration through the septa, secondary radiation generation and detector performance at small pixel size, the relatively large size of the apertures in the masks used in EI-BT (and consequently moderate aspect ratio) makes fabrication of thicker structures significantly easier, which is the key strategy to translate this type of imaging to higher X-ray energies. This is relevant and timely for betatron and other laser-driven X-ray sources since multi-petawatt laser facilities are expected to produce X-rays in the 0.1–10 MeV range (e.g., see ref. ^44^).

In conclusion, we have identified the source technology and the imaging technique that allow femtosecond multimodal imaging, designed and performed an experiment in which these have been effectively combined together, and demonstrated it is possible multimodal imaging and/or microscopy at the femtosecond time resolution. This result opens new avenues of application for laser-driven X-ray sources. To give an example, EI-BT experiments at synchrotrons have demonstrated that, in additive manufacturing, the nucleation points of droplet formation become visible in the scattering/phase channels much earlier than in attenuation^8^. Having the ability to follow the process with the same time resolution as the laser used for the manufacturing would enable a much deeper understanding and control of the process itself. More generally, femtosecond multimodal microscopy can advance ultrafast dynamic studies based on, e.g., pump and probe imaging, from laser ablation processes to 3D bioprinting^45^.

## Methods

### Laser-accelerator

The ALLS laser system can deliver 3.2 J, 22 fs at 2.5 Hz repetition rate. The laser pulse is linearly polarised and is focused with an F/15 off-axis parabola onto a supersonic gas jet well defined by a 7.5 mm long density plateau. In the focal plane, the FWHM spot size is 15 μm with 80% of the total energy contained within an area limited by the 1∕e^2^ radius. This corresponds to a laser intensity of 4.6 × 10^19^ W cm^−2^ and a normalised vector potential amplitude of *a*_0_ = 4.6.

### Sample/electrochemical cell preparation

Two electrochemical cells were prepared at the ALLS laboratory in an argon environment. The cells consist of two copper wires inside a 2 mm diameter Kapton capillary. The end of one of the two copper wires was covered with metal lithium, the other was left exposed. The gap in between the wires is 1.8 mm for the pristine cell and 1.1 mm for the deposited cell. The difference is due to the complexity of manufacturing. The cells were filled with electrolytes (1 M LiPF6 EC:DMC) and sealed with epoxy. During the process, an argon bubble was trapped inside the pristine cell. For the electrochemical cycling, a GAMRY 1010E potentiostat was used. For the lithium plating a constant current of 0.796 mA cm^−2^ (25 µA) was applied for ca. 9 h.

### Mask

The self-standing mask was manufactured by Scitech Precision Ltd via laser micromachining of a 50 μm thick tantalum foil. It consists of a vertical grating of 12 μm aperture and 39 μm period (accuracy +/−1 μm). The vertical lines are interrupted every 1.5 mm by horizontal and vertical bridges to reinforce the mechanical strength and avoid using a substrate. The total area of the mask is 1.5 × 1.5 cm. The mask is self-standing (frameless). A 2.7 × 3.1 mm cropped transmission image of the mask as recorded during the experiment is shown in Fig. 1c.

### Retrieval

The retrieval process consists of a line-by-line comparison of the mask intensity profile with and without the sample (see Fig. 1). The effects of the absorption, refraction and scattering induced by the sample on the line profile can be formulated as^46^:

$$I\left(x\right)=T{I}_{r}(x-\delta x)* s(x)$$

where ${I}$ and ${I}_{r}$ are the intensity profile with and without sample, *T* is the percentage transmission through the sample, $\delta x$ is the lateral shift of the beamlets at the detector plane due to refraction. The effect of the scattering due to the sample is represented by the convolution product with the sample scattering function $s(x)$.

The reference ${I}_{r}$ with no sample is assumed to be well represented by a Gaussian function:

$${I}_{r}=\frac{{A}_{r}}{\sqrt{2\pi {\sigma }_{r}^{2}}}\exp \left[-\frac{{\left(x-{\mu }_{r}\right)}^{2}}{2{\sigma }_{r}^{2}}\right]$$

Assuming the scattering $s(x)$ follows a normalised Gaussian distribution with standard deviation $\sigma$, then $I\left(x\right)$ can be expressed as:

$$I\left(x\right) =\frac{T{A}_{r}} {\sqrt{2\pi ({\sigma }_{r}^{2}+{\sigma }^{2})}}\exp \left[-\frac{ {\left(x-{\mu }_{r}-\delta x\right)}^{2}} {2({\sigma }_{r}^{2}+{\sigma }^{2})}\right]\\ =\frac{{A}_{s}} {\sqrt{2\pi {\sigma }_{s}^{2}}}\exp \left[-\frac{ {\left(x-{\mu }_{s}\right)}^{2}}{2{\sigma }_{s}^{2}}\right]$$

Gaussian interpolation of the experimental data can be used to determine transmission *T*, refraction *R* and scattering *S* as follow:

$$T=\frac{{A}_{s}}{{A}_{r}}$$

$$R=\frac{{\mu }_{s}-{\mu }_{r}}{z}$$

$$S=\frac{{\sigma }_{s}^{2}-{\sigma }_{r}^{2}}{{z}^{2}}$$

Where *z* is the sample to detector distance. The full mathematical derivation of the formulas above can be found in refs. ^7,47^.

For the analysis of the data presented here, the three-channel retrieval was performed using the Curve Fitting toolbox in Matlab. The images were split into vertical sections parallel to the grating lines containing 7 beamlets each. Each beamlet was approximated with a Gaussian function and the multiple fitting was performed with and without the sample.

Following the mathematical formulation above, the transmission channel was obtained as the ratio of the Gaussian amplitude with and without sample. The refraction channel is calculated as the shift of the Gaussian centres. The scattering is given by the difference in the variance of the Gaussian with and without the sample. When dithering was used, the 4 dithering images for each channel were then interlaced to give the full-resolution image.

For all the 100 and 50 multi-shot imaging, the original images were binned vertically by a factor of 2 before retrieval. for the single-shot image, the image was binned vertically by a factor of 8.

### Supplementary information

Supplementary Information

## Data Availability

The datasets generated during and/or analysed during the current study are available from the corresponding author upon reasonable request.
