# Supplementary material for: Femtosecond multimodal imaging with a laser-driven X-ray source
Source: Commun Phys. 2023 Oct 11;6(1):288. doi: 10.1038/s42005-023-01412-9 (PMC11041725; doi:10.1038/s42005-023-01412-9)
Supplement: Supplementary file 1 — Supplementary Information [file 42005_2023_1412_MOESM1_ESM.pdf]

Supplementary information for:

Femtosecond multimodal imaging with a laser-driven x-ray source

Adam Doherty[1**], Sylvain Fourmaux[2**], Alberto Astolfo[1], Ralf Ziesche[3], Jonathan Wood[4], Oliver Finlay[5], Wiebe Stolpe[6], Darren Batey[7], Ingo Manke[3], François Légaré [2], Matthieu Boone[6], Dan Symes[5], Zulfikar Najmudin[4], Marco Endrizzi[1], Alessandro Olivo[1], Silvia Cipiccia[1]*

[1] Department of Medical Physics and Biomedical Engineering, University College London, 2 Malet Pl, London WC1E 7JE, United Kingdom.
[2] Institut National de la Recherche Scientifique—Énergie, Matériaux et Télécommunications, Université du Québec, 1650 Lionel Boulet, Varennes J3X 1P7, Québec, Canada.
[3] Helmholtz-Zentrum Berlin für Materialien und Energie Hahn Meitner Platz 1, 14109 Berlin, Germany
[4] The John Adam Institute for Accelerator Science, Imperial College London, Prince Consort Rd, South Kensington, London SW7 2BW
[5] Central Laser Facility, Rutherford Appleton Laboratory, Harwell Campus, Didcot OX11 0QX, United Kingdom.
[6] UGCT-RP, Department of Physics and Astronomy, Ghent University, Ghent 9000, Belgium
[7] Diamond Light Source, Rutherford Appleton Laboratory, Harwell Campus, Didcot OX11 0QX, United Kingdom.

**Corresponding author: s.cipiccia@ucl.ac.uk*
***These authors contributed equally*

Supplementary Note 1:Mask:

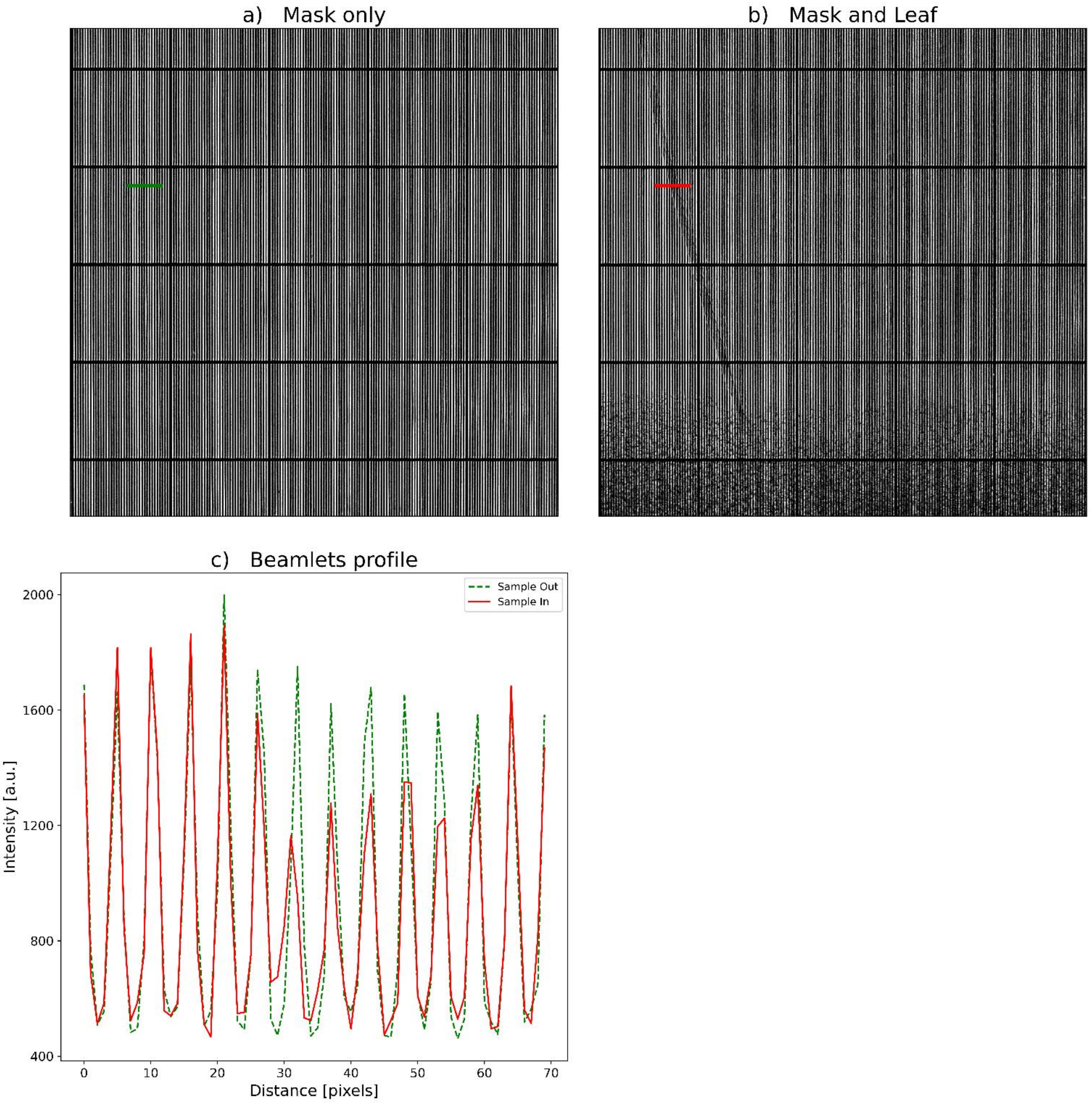

Figure S 1: EI-BT, raw data. A-b) An example raw images as acquired by the detector. a) Signal recorded by the detector when the mask is in and the sample out (reference). B) Signal recorded by the detector when both the mask and the sample are in. A) and b) are the average over 100 shots. C) beamlets profile with (solid red line) and without (reference-dashed green line) the sample extracted from the same location of a) and b) (green and red line respectively). By approximating the beamlets to gaussian and comparing the amplitude, centre position and width the transmission, refraction and scattering signals are retrieved.

Supplementary Note 2: Electron beam characterization:

The electron beam energy and charge were measured by diverting the electron beam into a calibrated Lanex screen using two consecutive permanent dipole magnets of 1.1 and 0.83 T respectively, each 10 cm long separated by a gap of 74 mm. This corresponds to an exit angle of 169 mrad at 400 MeV. The electron spectrometer energy range is from 140 MeV to 1 GeV. Full details on the electron spectrometer available at ALLS can be found in reference 1. During the experiment a maximum energy of 318 MeV and an average of 183 MeV, with a total charge of 685 pC were observed. A typical electron spectrum and divergence is shown in Figure S 2.

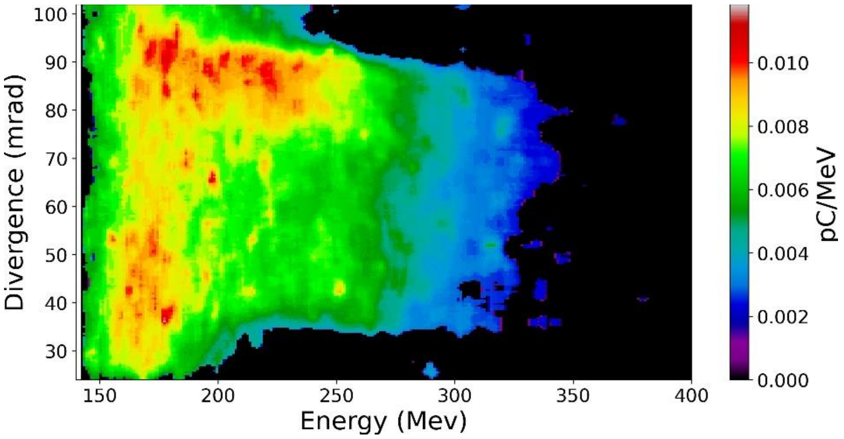

Figure S 2: Typical Electron Spectrum: Calibrated image of a typical electron spectrum and divergence.

In about 30% of the electron spectra, the signature of the betatron oscillations can be seen in the electron trajectories as shown in Figure S 3 a) raw image uncalibrated and b) calibrated for divergence and energy. It should be kept in mind that the electron beam that is measured may look very different to what it looked like when it was emitting most of the betatron x-rays.

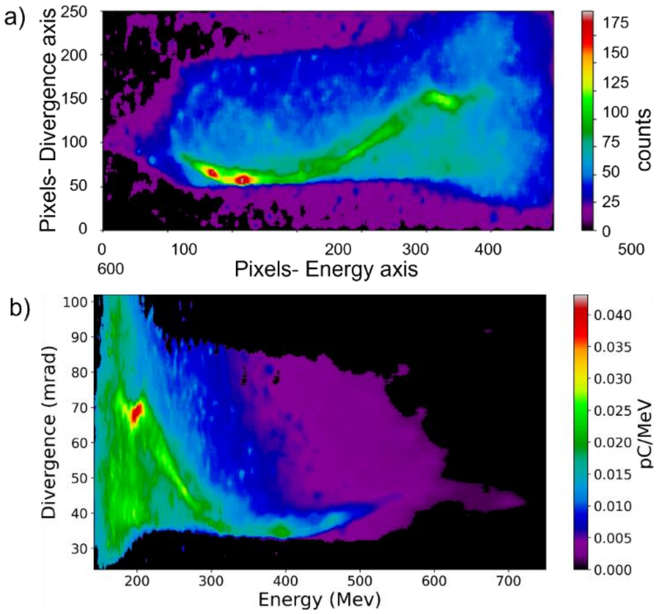

Figure S 3: Betatron signature on electron trajectories. A) raw image (uncalibrated) recorded by the camera looking at the electron spectrometer. B) same image calibrated in energy, charge and divergence. In both the betatron oscillations is visible.

Supplementary Note 3: Source size, position fluctuation, divergence and pointing effect on EI-BT

The realization of the EI principle does not require the use of a coherent beam: the method is in fact incoherent, fully achromatic[2] and non-interferometric, and can be described purely on the basis of x-ray refraction while neglecting any interference effect[3]. For this reason, assuming no angular dependence of the beam intensity profile and spectrum, the technique is not affected by changes in the divergence or pointing of the source. These indeed do not affect the position nor the shape of the shadow the mask casts on the detector (i.e. neither the position nor the shape of the beamlets).

Conversely, fluctuations in the source position will affect the position of the mask' shadow. The effect over multiple acquisitions is a linear sum over all source positions, which is equivalent to a correspondingly larger source, broadened by a factor corresponding to the standard deviation of the position fluctuation. How large a fluctuation (and therefore a source) the technique can cope with depends on the specific geometry.

Let us assume we have a point source at distance *m* from the mask of period *p* and aperture *a*, and *d* is the mask to detector distance (see schematic below). We call $(p-a)_1$the magnified shadow $(p-a)$ at the detector. This is given by:

$$(p-a)_1 = \frac{(p-a)}{m} * (m+d)$$

The maximum pointing fluctuation (or source size) that can be tolerated is determined by the position where the projected mask moves by half a period thus washing out the shadow (see Figure S 4), thereby causing excessive cross-talk between adjacent beamlets. From the geometrical sketch below this is given by:

$$\frac{\Delta S}{m} < \frac{(p-a)_1}{d}$$

In our geometry the limit is 58 μm in the horizontal direction, which is ten times larger than the measured σ_x.

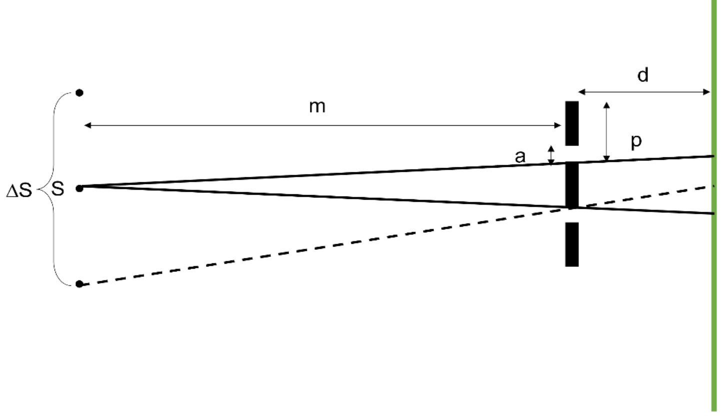

Figure S 4: Effect of source size/position fluctuation on EI-BT: Schematic based on ray tracing showing the limit on source size/position fluctuation to perform EI-BT as a function of the [illegible]

As a final note, the robustness of EI vs increasing source sizes has been discussed on many previous works[4,5] and imaging with focal spots of up to 100 micron is regularly carried out in standard laboratories[6].

Supplementary References:

[1] Fourmaux, S., Ta Phuoc, K., Lassonde, P., Corde, S., Lebrun, G., Malka, V., Rousse, A. & Kieffer, J. C. Quasi-monoenergetic electron beams production in a sharp density transition. Applied Physics Letters 101, (2012)

[2] Endrizzi, M., Vittoria, F. A., Kallon, G., Basta, D., Diemoz, P. C., Vincenzi, A., Delogu, P., Bellazzini, R. & Olivo, A. Achromatic approach to phase-based multi-modal imaging with conventional X-ray sources. Opt. Express 23, (2015). 16473-16480

[3] Diemoz, P. C., Endrizzi, M., Hagen, C. K., Millard, T. P., Vittoria, F. A. & Olivo, A. Angular sensitivity and spatial resolution in edge illumination X-ray phase-contrast imaging. Nuclear Instruments and Methods in Physics Research Section A: Accelerators, Spectrometers, Detectors and Associated Equipment 784, (2015). 538-541

[4] Olivo, A. & Speller, R. Modelling of a novel x-ray phase contrast imaging technique based on coded apertures. Phys Med Biol 52, (2007). 6555-6573

[5] Diemoz, P. C., Hagen, C. K., Endrizzi, M. & Olivo, A. Sensitivity of laboratory based implementations of edge illumination X-ray phase-contrast imaging. Applied Physics Letters 103, (2013)

[6] Astolfo, A., Buchanan, I., Partridge, T., Kallon, G. K., Hagen, C. K., Munro, P. R. T., Endrizzi, M., Bate, D. & Olivo, A. The effect of a variable focal spot size on the contrast channels retrieved in edge-illumination X-ray phase contrast imaging. Sci Rep-Uk 12, (2022). 3354
